# Investigating the association between yellow fever vaccination and symptomatic acute Zika virus infection: a case-control study

**DOI:** 10.1590/0037-8682-0233-2024

**Published:** 2025-06-02

**Authors:** Lisany Krug Mareto, Sahra de Almdeira Metzker, Camila Guadeluppe Maciel, Bruna Luiza de Amorin Vilharba, Fabio Antonio Venancio, Wagner de Souza Fernandes, Cláudia Du Bocage Santos-Pinto, Everton Falcão de Oliveira

**Affiliations:** 1 Programa de Pós-Graduação em Doenças Infecciosas e Parasitárias, Universidade Federal de Mato Grosso do Sul, Campo Grande, MS, Brazil.; 2 Faculdade de Medicina, Universidade Federal de Mato Grosso do Sul, Campo Grande, MS, Brazil.; 3 Residência Multiprofissional em Saúde da Família SESAU/FIOCRUZ, Secretaria Municipal de Saúde de Campo Grande, Campo Grande, MS, Brazil.; 4 Instituto de Biociências, Universidade Federal de Mato Grosso do Sul, Campo Grande, MS, Brazil.

**Keywords:** Cross protection, Vaccine, Yellow fever, Zika virus, Repositioning

## Abstract

**Background:**

During the Zika virus (ZIKV) epidemic in Latin America (2015-2016), a high incidence of microcephaly was observed in places with low yellow fever (YF) vaccination coverage. Cross-reactivity between ZIKV and YF virus was hypothesized as a possible explanation.

**Methods:**

We performed a case-control study to estimate the odds of ZIKV infection according to patients’ YF vaccination status. The study considered ZIKV fever as the outcome, and previous vaccination against YF as exposure. We included all confirmed cases of acute ZIKV fever in Campo Grande, Mato Grosso do Sul, from January 2015 to December 2018. The control group comprised age- and sex-matched residents of Campo Grande who had no history of acute febrile arboviruses during the study period.

**Results:**

The cumulative incidence of acute ZIKV fever was 1.87 cases per 1000 population. The case group had a predominance of women aged 20-39 years and white and mixed races. Vaccination coverage for YF was 65.5% in the case group and 60.9% in the control group. The odds ratio (OR) suggested a weak association between outcomes and exposure (OR = 1.23; 95% confidence interval [CI]: 1.05-1.44).

**Conclusions:**

Our findings provide no evidence that prior YF vaccination protects against acute ZIKV infection. Further studies are needed to analyze the development of anti-ZIKV and anti-YF neutralizing antibodies in affected individuals because new ZIKV epidemics are predicted.

## INTRODUCTION

Zika virus (ZIKV) is an arbovirus of the genus Flavivirus, belonging to the family Flaviviridae^1^, whose main vectors are the mosquitoes *Aedes aegypti* and *Aedes albopictus*, which are also vectors of dengue^2^, chikungunya^3^, and yellow fever (YF) viruses^4^. The main transmission route of ZIKV is vectorial^1^ ^,^ ^5^. However, non-vectorial routes of transmission have been reported, including sexual, transfusional, transplacental, and perinatal transmission during childbirth^5^ ^,^ ^6^.

Approximately 50 years after the identification of the first cases of acute febrile illness caused by ZIKV in Africa and Asia^1^, two epidemics were recorded in 2007 and 2013 in the Pacific islands of Oceania^7^. In Latin America, the first cases were recorded in 2014 and the epidemic peaked in 2015-2016, with Brazil being the most affected country^8^. Between 2015 and 2020, 215,327 cases were reported in the country, and the disease incidence was 1.03 cases per 100,000 inhabitants, with the Midwest and Northeast regions being the most affected^9^.

Acute ZIKV fever is a self-limiting disease similar to dengue fever. The symptoms are usually mild and may include maculopapular rash, conjunctivitis, low-grade fever, myalgia, arthralgia, and headache^5^ ^,^ ^6^. However, other severe outcomes related to vertical transmission of ZIKV have been observed in newborns, including congenital ZIKV syndrome (CZS)^10^ ^,^ ^11^. Severe neurological outcomes, such as Guillain-Barré syndrome^12^ and the possibility of long-term neurologic effects^13^ have been reported in adults.

ZIKV has the same genomic organization as other viruses of the genus Flavivirus, such as viruses that cause YF, dengue fever, Japanese encephalitis, and West Nile virus fever^14^. These viruses share some surface structures with the viral envelope and capsid, especially the E and C proteins, which have important functions during immunological recognition by the host^14^ ^,^ ^15^ and act as primary targets of neutralizing antibody-mediated responses^16^. Owing to this phylogenetic similarity, cross-reactions between viruses of the same family are expected, and some authors have suggested that previous dengue fever or ZIKV exposure can drive infection enhancement or neutralization of other flaviviruses^17^ ^-^ ^21^.

During the ZIKV epidemic in Latin America, regions with low YF vaccination coverage, such as the Northeast region^21^, where routine vaccination was not recommended until 2020^22^, exhibited a high incidence of microcephaly^23^. Conversely, areas with pre-2015 recommendations for YF vaccination, and consequently higher vaccination coverage, displayed a lower incidence of microcephaly due to congenital ZIKV syndrome^24^. Considering the hypothesis that pregnant women residing in areas with high YF vaccination coverage may have offspring with a reduced risk of microcephaly^22^, and that pre-existing immunity to co-circulating flaviviruses acquired through either natural infection or vaccination could influence the severity of ZIKV infection and its impact on pregnancy outcomes^25^, it is plausible to hypothesize that YF vaccination might confer protection against symptomatic acute ZIKV infection in the general population. This protection could potentially mitigate the risk of severe ZIKV-related outcomes, primarily associated with symptomatic diseases. 

Drug repurposing is a strategy that can be explored during public health emergencies, and the possibility of repositioning the YF vaccine to protect against ZIKV infection has been raised by some authors^16^. Drug repositioning strategies use drugs already approved and marketed or still under investigation but with a safety profile already demonstrated in clinical trials for the treatment of other conditions^26^. Studies have been conducted to test the Bacillus Calmette-Guérin vaccine as a protective factor against coronavirus disease 2019, with the justification that the vaccine would promote a general increase in long-term immunological mechanisms^27^ and thus would reduce the incidence and severity of severe acute respiratory syndrome coronavirus 2 infection^28^.

Concerning ZIKV, the possibility of repositioning the immunobiological effect of the YF vaccine could be due to cross-reactivity^29^ between the YF vaccine and ZIKV, which is explained by the presence of the envelope gene (E) and the non-structural protein (NS5) in both viruses, and by the cell-mediated immune response and non-neutralizing antibody^16^, since cross-protection or enhancement can also occur because of T cells. NS5 is a conserved and multifunctional protein that constitutes the viral polymerase and is present in the attenuated YF vaccine. This similarity motivated studies to test the cross-reactivity between YF vaccine recipients and ZIKV-specific antigens^16^.

Currently, there are no specific treatments or licensed vaccines for preventing ZIKV infection, although research on the development of vaccines and other preventive measures is ongoing^30^. The ZIKV epidemic and its serious outcomes have caused public emergency, making the emergence of new epidemics possible^20^ ^,^ ^31^. This study aimed to verify the association between previous vaccinations against YF and the occurrence of symptomatic forms of ZIKV. 

## METHODS

### Study design, setting, and period

In this matched (age and sex) case-control study, ZIKV fever (symptomatic infection) was the outcome and previous YF vaccination was the exposure. This study, based on secondary data obtained from official databases, was conducted in the city of Campo Grande, Mato Grosso do Sul. Cases diagnosed and reported to the Brazilian Notifiable Diseases Information System (SINAN) between January 2015 and December 2018 were assessed for this study.

Campo Grande, the capital of the state of Mato Grosso do Sul, had an estimated population of 885.711 inhabitants in 2018^32^. Of this total, it was estimated that 55.2% lived in areas covered by primary care in the period between 2014 and 2018^33^.

Two groups were formed: the case group comprised individuals with confirmed diagnoses of ZIKV acute fever and the control group comprised individuals with no history or notification of acute ZIKV fever, dengue fever, or chikungunya.

### Case and control definitions

In this study, we defined a confirmed case of acute fever as that described by the Brazilian Ministry of Health. Every suspected case (characterized by a pruritic maculopapular rash accompanied by at least one of the following: fever, conjunctival hyperemia or non-purulent conjunctivitis, arthralgia/polyarthralgia, or periarticular edema) was confirmed through laboratory investigations, including viral isolation, detection of viral RNA via reverse transcriptase-polymerase chain reaction (RT-PCR), and IgM serology^34^.

Cases reported to the SINAN, the official system for registering notifiable diseases in Brazil that met the criteria for confirmed cases, were included in the study. Due to the unavailability of specific laboratory confirmation methods in the notification form, it was not possible to determine the exact laboratory procedures used. From 2015 to 2018, 1626 confirmed cases of ZIKV fever were reported in Campo Grande, all of which were included in the case group of this study.

The control group was formed using a 1:1 ratio and matched for age (plus or minus 5 years) and biological sex assigned at birth. A list of possible controls was obtained from the Primary Care Information System Database and the nominal list of all visits to primary and outpatient care in Campo Grande between 2014 and 2018. Because Zika acute fever has a differential diagnosis from two other arboviruses, individuals who were reported in SINAN with suspected or confirmed cases of ZIKV fever, dengue fever, or chikungunya were excluded from the list of possible controls. For each enrolled patient, one control was selected from the database.

The public health management system, used by the Municipal Health Secretary of Campo Grande (SESAU-Hygia), was used to verify the vaccination status (until 2014 - 1 year prior to the beginning of the ZIKV epidemic in Brazil) of all individuals included in the study against YF, regardless of the number of doses administered. Cases and controls who were not actively registered at the SESAU-Hygia were excluded from the study.

Table 1 describes the variables evaluated in this study based on the data collection sources.

Table 1 describes the variables evaluated in this study based on the data collection sources.

**TABLE 1:** Study data classified according to groups.

Variable and data source	Case group	Control group
Socioeconomic and demographic data: SINAN, SISAB e-SUS		
Municipality of residence	X	
Notification date	X	
Date of first symptoms	X	
Birth date	X	X
Age	X	X
Sex	X	X
Race/skin color	X	
Education	X	
Address	X	
Zone (rural or urban)	X	
Final classification of the disease	X	
Case evolution	X	
Vaccination Status: SESAU-Hygia		
Record of administered dose	X	X
Number of doses administered	X	X
Date of dose(s) administered	X	X

**SINAN:** Brazilian Notifiable Diseases Information System; **SISAB:** Primary Care Information System Database; **e-SUS:** electronic medical record of the Brazilian Unified Health System; **SESAU-Hygia:** Municipal Health Secretary of Campo Grande.

### Data analyses

Descriptive statistics were used to characterize the study population (mean, standard deviation, median, minimum, maximum, percentile, and percentage). The proportion of participants who were vaccinated against YF before the beginning of the ZIKV epidemic in Brazil was used as a measure of the frequency of exposure in both groups. Odds ratio (OR) was used as a measure of association for comparisons between groups. The Mantel-Haenszel odds ratio was used to assess confounding factors in the stratified analysis by sex and age group.

A significance level of 5% was adopted for all hypothesis tests, and confidence intervals (CIs) were calculated at 95%. Analyses were performed using R software version 4.4.0 (R Foundation, Vienna, Austria) and the following packages were used: descr^35^ and epiR^36^.

### Ethical consideration

This study was approved by the Research Ethics Committee of the Federal University of Mato Grosso do Sul (CAAE 38010020.4.0000.0021) under protocol nos. 4,313,059. The Research Ethics Committee of the Federal University of Mato Grosso do Sul waived the requirement for informed consent because of the anonymization of data. The data for this study were accessed for research purposes starting on November 1, 2020, and ending on June 30, 2021. During data collection, the researchers had access to participants' names only to verify their vaccination status in the SESAU-Hygia system. Immediately afterward, the names were replaced with the codes used in the study.

## RESULTS

During the study period, 1626 confirmed cases of ZIKV infection were reported in Campo Grande, corresponding to an accumulated incidence of 1.87 cases per 1000 inhabitants. Over the time series evaluated, 2016 was the year with the highest number of notifications of confirmed cases, totaling 1413 (86.9%), which represented an incidence of 1.63 cases per 1000 inhabitants (Table 2).

The mean age was 33.7 years (median = 32; standard deviation = 16.4), with the most prevalent age groups being 3039 years (24.1%) and 20 to 29 years (23.6%). Cases were more frequent (73.7%) among women. Regarding race/skin color, there was a predominance of whites and browns with 48.8% and 28.7% of notifications, respectively. Most participants had an elementary education (38.8%); high school and higher education were reported by 13.4% and 6.6% of individuals, respectively. Data on schooling were unavailable for 41.1% remainder of the participants. Most of patients with confirmed cases recovered without serious outcomes (94.7%), and no deaths were recorded as a result of ZIKV infection (Table 3).

The control group included 1626 individuals who were randomly sampled by simple drawing and matched with the cases. The mean age of the control group was 33.8 years (median = 32; standard deviation = 17.4), with the predominant age groups being 20 to 29 years old (24.48%) and 30 to 39 years old (23.00%) (Table 3).

**TABLE 2:** Incidence of acute Zika virus fever per 1000 inhabitants, Campo Grande, 2015-2018.

Year of Notification	n	%	Inhabitants	Incidence
2015	116	7.1	853,622	0.04
2016	1413	86.9	863,982	1.63
2017	26	1.6	874,210	0.02
2018	71	4.4	885,711	0.08
**Total**	**1626**	**100.0**	-	-

**TABLE 3:** Sociodemographic data of cases of Zika virus acute fever (case group) and control group, Campo Grande, 2015-2018 (n = 1626).

Group	Case	Control
Variable	n (%)	n (%)
**Age (years)**		
0-9	109 (6.7)	124 (7.6)
10-19	169 (10.4)	181 (11.1)
20-29	384 (23.6)	400 (24.6)
30-39	391 (24.1)	375 (23.1)
40-49	247 (15.2)	240 (14.8)
50-59	194 (11.9)	189 (11.6)
≥60	132 (8.1)	117 (7.2)
**Sex**		
Man	1198 (73.7)	1199 (73.7)
Woman	428 (26.3)	427 (26.3)
**Race/skin color**		
Yellow	25 (1.5)	-
White	794 (48.8)	-
Indigenous	3 (0.2)	-
Browns	391 (28.7)	-
Black	247 (2.6)	-
Not informed	294 (18.1)	-
**Education**		
Incomplete elementary school	278 (17.1)	-
Complete elementary school	78 (4.8)	-
Incomplete high school	123 (7.6)	-
Complete high school	218 (13.4)	-
Incomplete higher education	47 (2.9)	-
Complete higher education	63 (3.9)	-
Not informed	738 (45.4)	-
Not applicable (<6 years old)	81 (5.0)	-
**Case evolution**		-
Cure	1540 (94.7)	-
Death	0 (0.0)	-
Not informed	86 (5.3)	-
**Total**	**1626 (100.0)**	**1626 (100.0)**

After checking the eligibility of all the recruited individuals, 688 were excluded because their data were not available in the SESAU-Hygia. Data unavailability made it impossible to check the vaccination status of 312 individuals (19.2%) in the case group and 376 (23.1%) in the control group. Therefore, association measures were estimated with only 1314 individuals in the case group and 1250 in the control group.

After excluding individuals with missing data, the mean age of the case group was 33.8 years (median = 32; standard deviation = 16.4), with the most prevalent groups aged 30 to 39 years (326; 24.8%). and 20 to 29 years (323; 24.6%) and 40 to 49 years (185; 14.1%). Women represented 75.7% (994) of the cases, and men represented 24.3% (320) of cases. In the control group, the mean age was 32.7 years (median = 31; standard deviation = 17.7), with the most prevalent groups aged 20 to 29 years (24.6%) and from 30 to 39 years old (23.0%). Women represented 74.9% of the cases, whereas men represented 25.1% of the cases.

In both groups, 1620 individuals were vaccinated for YF by the year 2014, the year before the ZIKV epidemic in Latin America: 861 (65.5%) in the case group and 759 (60.9%) in the control group. In the case group, 737 (26.1%) received one dose of the vaccine, 121 (9.2%) two doses and 3 (0.2%) received three doses. Among the controls, 635 (51.0%) received the first dose, 120 (15.9%) two doses and (0.5%) received 3 doses (Table 4).

**TABLE 4:** Yellow fever vaccination coverage.

	Case group	Control group
	n (%)	n (%)
**Vaccination status**		
Vaccinated	861 (65.5)	759 (60.7)
Unvaccinated	453 (33.1)	491 (39.2)
**Administered doses**		
1	737 (56.1)	635 (50.8)
2	121 (9.2)	120 (9.6)
3	3 (0.2)	4 (0.3)
**Total**	**1314 (100.0)**	**1250 (100.0)**

Table 5 presents the measures of association for the occurrence of acute ZIKV fever according to vaccination status, including stratification by sex and age group. The crude OR for being previously vaccinated against YF among those with ZIKV fever was 1.23 (95% CI 1.05-1.44). After adjusting for confounders through stratification by sex, the Mantel-Haenszel OR remained at 1.23 (95% CI 1.05-1.44), indicating that prior YF vaccination was associated with a 23% increase in the odds of developing ZIKV fever. For age-stratified analysis, the adjusted Mantel-Haenszel OR was 1.23 (95% CI 1.05-1.45), suggesting a similar association across different age groups. These results suggest a weak association between prior YF vaccination and increased odds of developing symptomatic ZIKV fever, as the OR values were close to 1, indicating near non-association.

**TABLE 5:** Odds ratio for the occurrence of ZIKV fever in study participants vaccinated and unvaccinated against yellow fever.

General	Case	Control	OR (95% CI)	OR MH (95% CI)
Vaccinated	861	759	1.23 (1.05, 1.44)	
Unvaccinated	453	491		
**Sex**				1.23 (1.05, 1.44)
**Woman**				
Vaccinated	659	569	1.27 (1.05, 1.53)	
Unvaccinated	335	367		
**Man**				
Vaccinated	202	190	1.12 (0.81, 1.54)	
Unvaccinated	118	124		
**Age (years)**				1.23 (1.05, 1.45)
**0-19**				
Vaccinated	160	164	1.47 (1.01, 2.14)	
Unvaccinated	69	104		
**20-59**				
Vaccinated	631	534	1.21 (1.01, 1.46)	
Unvaccinated	351	360		
**≥60**				
Vaccinated	70	61	0.94 (0.51, 1.73)	
Unvaccinated	33	27		

**OR:** odds ratio; **CI:** confidence interval.

## DISCUSSION

Although the incidence of ZIKV infection has declined dramatically worldwide^7^, the outcomes associated with this infection remain a public health problem^37^. The present study investigated the association between previous vaccination for YF and the occurrence of symptomatic acute ZIKV infection.

Few clinical or epidemiological studies have investigated this relationship in humans^16^. Thus far, this is the first study conducted in the Midwest region of Brazil, one of the regions that had the highest incidence of the disease during the epidemic that occurred in Brazil^9^ but had a low incidence of microcephaly when compared to the Northeast region^21^ ^,^ ^22^ ^,^ ^24^.

As observed in other regions of Latin America that have reported ZIKV epidemics^7^, the highest peak occurred in Campo Grande in 2016. The epidemiological profiles of these cases were similar to those observed in other locations^5^ ^,^ ^8^ ^,^ ^38^. Notably, there is a higher frequency of cases in women; given the clinical repercussions of infection, including CZS, pregnant women are expected to have greater demand for health services in the presence of signs and symptoms of febrile illnesses.

In our study, the vaccination coverage for YF was similar between the two groups, with approximately two-thirds of the population vaccinated. However, the OR indicated a weak association between previous vaccinations and the occurrence of ZIKV acute fever, contradicting the hypothesis that the vaccine could provide protection against the illness^21^ ^,^ ^28^. However, the lower limits of the confidence intervals were close to 1, indicating no association. Moreover, Brady et al.^11^ found no evidence that the risk of microcephaly was affected by concurrent dengue fever or previous YF vaccination. Thus, there is no evidence that vaccination against YF provides protection against severe forms of ZIKV infection such as microcephaly.

Although the findings of the present study do not confirm our hypothesis, it is important to investigate this scenario and the vaccine itself further through experimental studies. The development of new vaccines is a strategy promoted by public health policies; however, this process can take many years, a period in which the population is exposed to the risk of illnesses^27^. Drug repositioning is considered an important strategy, especially in the case of neglected diseases for which investments in research and development are not considered advantageous in terms of the market^39^. Thus, the existence of an already-used vaccine with the potential to prevent a second condition needs to be explored, as it would preserve numerous resources and save lives.

Given that ZIKV typically manifests as a mild illness in adults, but can lead to severe outcomes, YF vaccination could potentially provide additional protection for women residing in regions with concurrent YF and ZIKV circulation. Although our study did not confirm the initial hypothesis that YF vaccination protects against symptomatic ZIKV infection, it must be noted that this was a local study and our findings contrast with those reported in other studies^21^. Regardless, YF vaccination has significant public health benefits, particularly in regions where YF is endemic. Therefore, the recommendations for YF vaccination in young women of reproductive age, especially in areas with concurrent YF and ZIKV transmission, should be supported. Further research is required to definitively evaluate the potential indirect benefits of YF vaccination in mitigating ZIKV disease severity, especially during pregnancy.

The limitations of this study include the quality of the data itself, as case notifications and YF vaccination statuses were completed by health professionals in both primary care and hospital settings, making them subject to the actions and accuracy of these individuals. In addition to the limitations related to the actions of healthcare professionals, the immunization registration platform, which manages computerized immunization records, is not a unified system. Consequently, individuals from other states or even cities may not have their vaccinations recorded unless they present their physical vaccination cards, where vaccines are documented by healthcare professionals. This limitation affects the verification of the vaccination status, potentially leading to underreporting or errors in the recording of information^40^. Additionally, it must be considered that the approach to data collection adopted in this study precludes the verification of post-vaccination seroconversion to YF, since the registration of the administered dose does not imply the development of a vaccine response^41^. Literature indicates percentages close to 98% of post-vaccination seroconversion with the 17DD-YFV (YF) vaccine^42^ ^,^ ^43^ ^,^ ^44^. However, there are reports of a decline in immunity over time^45^, which points to the need for further studies to quantify anti-YF neutralizing antibodies in people with ZIKV infection, together with a control group. Finally, it is important to highlight that this study was carried out in the Brazilian capital using an official database that concerns the entire population residing in the municipality. Although underreporting may exist, these are important results that make it possible to reflect on new steps and directions for ZIKV research and control strategies.

This study aimed to test the hypothesis that the yellow virus vaccine could protect against ZIKV infection, which has serious consequences in the Brazilian population. We found no evidence that prior YF vaccination protected against acute ZIKV infections. Thus, studies that perform serological analyses in individuals infected with ZIKV are needed, as they would allow observation of the development of acquired immunity and antibodies in patients. Furthermore, epidemiological studies in regions with high rates of infection and low vaccination coverage for YF, such as Northeast Brazil, could add to the results of the present study to elucidate whether the YF vaccine could act as a protective factor against ZIKV infections.
